# Germline Genomic and DNA Methylation Differences Between MGUS and Multiple Myeloma Among Patients of African Ancestry

**DOI:** 10.3390/cells15191722

**Published:** 2026-09-22

**Authors:** Jing Han, Sophie Hongfei Liu, Shahram Arsang-Jang, Zongli Xu, Kelly E. Rentscher, Angela J. Mathison, Mike Tschannen, Fumou Sun, Paul L. Auer, Sarah Kerns, Parameswaran Hari, Binod Dhakal, Siegfried Janz, Victor X. Jin, Raul Urrutia, Anita D’souza, Jing Dong

**Affiliations:** 1 Division of Hematology and Oncology, Department of Medicine, Medical College of Wisconsin, 8701 Watertown Plank Road, HRC 5860, Milwaukee, WI 53226, USA; 2 Department of Science & Technology, Jiangsu Cancer Hospital, Jiangsu Institute of Cancer Research, The Affiliated Cancer Hospital of Nanjing Medical University, Nanjing 210008, China; 3 Mellowes Center for Genomic Sciences and Precision Medicine, Medical College of Wisconsin, Milwaukee, WI 53226, USA; 4 Biostatistics and Computational Biology Branch, National Institute of Environmental Health Sciences, National Institutes of Health (NIH), Research Triangle Park, NC 27709, USA; 5 Department of Psychiatry and Behavioral Medicine, Medical College of Wisconsin, Milwaukee, WI 53226, USA; 6 Medical College of Wisconsin Cancer Center, Milwaukee, WI 53226, USA; 7 Division of Research, Department of Surgery, Medical College of Wisconsin, Milwaukee, WI 53226, USA; 8 Division of Biostatistics, Data Science Institute, and Cancer Center, Medical College of Wisconsin, Milwaukee, WI 53226, USA; 9 Department of Radiation Oncology, Medical College of Wisconsin, Milwaukee, WI 53226, USA; 10 Department of Molecular Pharmacology and Experimental Therapeutics, Mayo Clinic, Phoenix, AZ 85054, USA

**Keywords:** multiple myeloma, monoclonal gammopathy of undetermined significance, whole-exome sequencing, methylation

## Abstract

Individuals of African ancestry (AA) have approximately twofold higher risk of multiple myeloma (MM) than individuals of European ancestry, yet the molecular determinants between monoclonal gammopathy of undetermined significance (MGUS) and MM remain poorly defined. We performed whole-exome sequencing (WES) and genome-wide DNA methylation profiling in 28 AA patients with MM and 28 frequency-matched AA patients with MGUS without documented progression. After quality control, the WES analysis included 28 patients with MGUS and 27 with MM, while the DNA methylation analysis included 27 patients with MGUS and 26 with MM. Whole-exome sequencing identified differences in observed germline variant distributions, with qualifying *COL19A1* variants observed only in MGUS and qualifying *VWA5B2* variants observed only in MM within this cohort. Gene-burden analyses identified nominal exploratory signals in *LAMA5* and *PIEZO1*, although neither remained significant after Bonferroni correction. DNA methylation profiling identified 703 differentially methylated positions and 122 exploratory candidate differentially methylated regions between MM and MGUS. One 395 bp region spanning 16 CpG sites within *RNF39* met the FWER significance threshold. Exploratory pathway analyses implicated oncogenic signaling, cellular stress responses, and cell-death pathways. Although epigenetic age acceleration did not differ between groups, lower DNA methylation (DNAm)-estimated telomere length was observed in MM than in MGUS; however, this difference was borderline after adjustment for DNAm-estimated blood-cell composition. Together these exploratory findings identify germline and epigenetic features associated with MM versus MGUS in AA patients and nominate biological pathways for validation in larger, longitudinal studies.

**To the Editor:**

Multiple myeloma (MM) is an incurable plasma cell malignancy that disproportionately affects individuals of African ancestry (AA). Compared with individuals of European ancestry (EA), AA individuals have approximately twofold higher lifetime risk of both MM and its precursor condition, monoclonal gammopathy of undetermined significance (MGUS) [1]. These disparities may reflect a complex interplay of genetic, social, environmental, healthcare-related, and other factors that remain incompletely understood [2,3]. Genome-wide association studies (GWAS) comparing patients with MM with population controls have identified 35 susceptibility loci [4]; however, nearly all studies have been conducted in populations of EA. The only AA-focused GWAS reported to date did not identify novel risk loci [5], leaving the contribution of inherited genetic variation to MM among AA individuals incompletely characterized. We previously identified 13 nuclear and mitochondrial loci associated with the risk of MM compared with MGUS [6], suggesting that inherited genetic variation may distinguish the malignant and precursor disease states. Given the greater genomic heterogeneity in AA individuals, rare coding variants may contribute to genetic differences that are not captured by common-variant GWAS [7,8]. Because disease-associated variants are enriched in coding regions and are often rare and population-specific, whole-exome sequencing (WES) provides a complementary approach for identifying potentially functional germline variants.

In addition to germline variation, epigenetic dysregulation has been reported across MGUS and MM disease states. Aberrant methylation involving CDKN2A (p16), DAPK1, and CDH1 (E-cadherin) has been observed in studies comparing MGUS and MM [9,10,11], and genome-wide studies have identified broader disruption of DNA methylation in plasma-cell disorders [12]. Blood-based DNA methylation profiles can also be used to estimate biological-aging measures, including epigenetic age and DNA methylation-derived telomere length, which have been studied in relation to cancer phenotypes [13]. However, blood-based DNA methylation differences between MM and MGUS remain poorly characterized among AA patients.

Here, we performed WES and genome-wide DNA methylation profiling in 28 AA patients with MM and 28 AA patients with MGUS who had no documented progression to MM during at least five years of follow-up. The median follow-up from MGUS diagnosis was 11.7 years (range, 5–22 years). We sought to identify rare, predicted deleterious germline variants and DNA methylation features associated with MM versus MGUS without progression during the observed follow-up period. This study was approved by the Institutional Review Board at the Medical College of Wisconsin (MCW) and conducted in accordance with the Declaration of Helsinki. Participants were frequency matched between MM and MGUS groups by age (±5 years), sex, and immunoglobulin isotype. The median age at diagnosis was 65 years (range, 46–78) in the MGUS group and 64 years (range, 42–79) in the MM group. Each group included nine male patients (32.1%). All participants were Black and had genetically inferred African ancestry. Immunoglobulin isotype distributions were identical between groups: 24 patients (85.7%) had IgG, three (10.7%) had IgA, and one (3.6%) had a light-chain isotype in each group (Supplementary Table S1). Detailed methods are provided in the Supplementary Methods. Briefly, WES was performed on buffy coat DNA using the Illumina DNA Prep with Enrichment kit (Illumina, Inc., San Diego, CA, USA) and sequenced on the Illumina NovaSeq 6000 platform (2 × 150 bp; mean target coverage, 100×). Following quality control (QC), 55 individuals (28 MGUS and 27 MM) were included in genetic analyses. After variant filtering and functional annotation using Ensembl Variant Effect Predictor version 115, 223,104 rare variants with a gnomAD allele frequency ≤ 1%—prioritizing the African/African American population frequency—were identified. Of these, 19,882 met the predicted-deleteriousness criteria, defined as protein-truncating variants; missense variants classified as deleterious by SIFT (score ≤ 0.05) or as possibly or probably damaging by PolyPhen-2 (score > 0.446); or variants classified as pathogenic or likely pathogenic by CharGer. Qualifying variants included missense (88.8%), frameshift (4.0%), splice-site (3.2%), stop-gained (3.2%), and start- or stop-lost (0.9%) variants. Single-variant analyses were restricted to predicted deleterious rare variants carried by at least three participants to reduce extremely sparse comparisons and unstable effect estimates. Thirty variants were nominally associated with MM versus MGUS (*p* < 0.05, Supplementary Table S2).

For descriptive analyses, a rare coding variant was classified as “group-observed-only” if it was detected in at least one participant in one study group but not in any participant in the other group. A gene was classified as “group-observed-only” if its qualifying rare coding variants were detected in only one study group and no qualifying variant in that gene was detected in the other group. These classifications refer solely to observations within the analyzed cohort and do not imply biological specificity to MGUS or MM (Supplementary Methods and Supplementary Table S3). Among variants observed only in the MGUS group, rare, predicted deleterious coding variants were most frequently found in *COL19A1* (eight variants), followed by *KIF27* and *MUC22* (seven each) (Figure 1A). *COL19A1* encodes an extracellular matrix protein involved in tissue structure and cell–matrix interactions. Prior familial MM studies in EAs have reported rare pathogenic germline variants in *COL19A1* [14], suggesting a potential shared germline contribution across precursor and malignant stages. Among variants observed only in the MM group, rare coding variants were most frequently found in *VWA5B2* (eight variants), followed by *OXCT2* and *OTOGL* (six each) (Figure 1B). Although the role of *VWA5B2* in MM is unknown, its predicted function in cell adhesion and extracellular matrix interactions suggests potential relevance to plasma cell biology [15]. Given the small group sizes and low variant frequencies, the apparent restriction of these variants to one group may reflect sparse sampling and should not be interpreted as evidence of group-specific genetic associations. Burden analyses of shared genes identified several nominal associations with MM versus MGUS without documented progression during the observed follow-up period (*p* < 0.05), although none remained significant after Bonferroni correction (Supplementary Table S4). Among the nominal exploratory signals, *LAMA5* showed the strongest inverse association with MM (OR = 0.32, *p* = 2.5 × 10^−3^), whereas PIEZO1 showed the strongest positive association (OR = 7.26, *p* = 4.4 × 10^−3^); however, neither association remained significant after Bonferroni correction. *LAMA5* encodes a key extracellular matrix component involved in cell adhesion and microenvironmental signaling, while *PIEZO1* encodes a mechanosensitive ion channel implicated in calcium signaling, cellular proliferation, and tumor progression [16]. Exploratory gene-set enrichment analysis using a permissive FDR threshold of *q* < 0.25 identified candidate enrichment of PDGF signaling, basophil-associated immune programs, and early stress/proliferation pathways in MM, whereas a dendritic cell immune response signature was enriched in MGUS (Supplementary Table S5), suggesting potential differences in immune and stromal signaling between the groups. Collectively, these exploratory findings suggest that germline variation in immune regulation and microenvironment-related pathways may differentiate MM from MGUS in AA patients. Given the small sample size and lack of associations surviving multiple-testing correction, these candidate signals require validation in larger, independent cohorts.

Genome-wide DNA methylation profiling was performed using the Illumina Infinium HumanMethylationEPIC v2.0 BeadChip. After QC, 53 individuals (27 MGUS and 26 MM) were included. Using thresholds of q ≤ 0.05 and |Δβ| ≥ 0.1, we identified 703 differentially methylated positions (DMPs) between MM and MGUS (Supplementary Table S6). Of these, 166 were hypermethylated and 537 were hypomethylated in MM relative to MGUS. Most DMPs were located in intronic regions (50.1%), followed by intergenic regions (28.7%), promoter–transcription start site regions (11.5%), and exonic regions (4.3%). The remaining DMPs were located in transcription termination site regions (2.1%), 3′ untranslated regions (1.8%), other noncoding regions (1.0%), and 5′ untranslated regions (0.4%) (Supplementary Figure S1). Exploratory pathway analysis of genes associated with the 703 DMPs was performed using QIAGEN Ingenuity Pathway Analysis (IPA). The top nominally enriched canonical pathways included KIT- and FLT3-associated growth factor signaling, VEGF-mediated angiogenic signaling, FOXO-regulated oxidative stress and metabolic responses, ribonucleotide reductase signaling related to DNA replication and repair, and TP53/BRCA1/MDM4-associated signaling. Upstream regulator analysis bioinformatically inferred regulatory signatures associated with dexamethasone, ERBB2, β-estradiol, TGFB1, and FGF2. Collectively, these exploratory findings implicated growth factor-mediated RTK–RAS/PI3K signaling, cellular stress responses, and hormone-responsive regulatory networks.

Differentially methylated regions (DMRs) between MM and MGUS were identified using *bumphunter*, with a minimum absolute β-value difference of 0.05 and 1000 permutations. The DMR associated with *RNF39* (ring finger protein 39) was the only region meeting the significance criterion of a family-wise error rate (FWER) ≤ 0.05 (FWER = 0.008; area-level FWER [fwerArea] = 0.029). This 395 bp region contained 16 CpG sites and spanned exon 4 and part of the adjacent intron 3 of *RNF39*. The regional β-value difference was 0.156, corresponding to 15.6 percentage points higher methylation in MM than in MGUS. *RNF39* encodes a RING-type E3 ubiquitin ligase implicated in protein ubiquitination and innate immune signaling. Differential methylation at the *RNF39* locus has previously been reported in several nonmalignant conditions, including multiple sclerosis [17], post-traumatic stress disorder [18], and intellectual disability [19]. However, to our knowledge, differential methylation of *RNF39* has not previously been reported in MGUS or MM. The functional relevance of this region to plasma-cell disease remains to be determined. For hypothesis generation, 122 candidate regions meeting both FWER < 1 and an area-based *p* value (*p*.valueArea) ≤ 0.025 were prioritized for exploratory pathway analysis (Supplementary Table S7). These regions were not considered statistically significant DMRs. Their genomic distribution was broadly similar to that of the DMPs, with most located in intronic and intergenic regions, followed by promoter–TSS regions (Supplementary Figure S2). Exploratory analysis of genes associated with these 122 candidate regions identified nominal enrichment of p53 signaling, pyroptosis, and intrinsic apoptosis pathways. IPA also bioinformatically inferred an EP300-centered transcriptional network involving *RNF39*, *TP53*, *FOXP1*, and *SMAD6* (Supplementary Figures S2 and S3). EP300 is a histone acetyltransferase that regulates enhancer activity and cellular stress-response transcription through H3K27ac-mediated chromatin remodeling [20]. Because the IPA *p* values were not adjusted for multiple testing, these findings should be considered hypothesis-generating. Collectively, the exploratory DMP- and DMR-based analyses implicated growth and survival signaling, cellular stress responses, DNA damage and repair, apoptosis, and transcriptional regulation in MM versus MGUS without documented progression during the observed follow-up period. Future studies could validate prioritized CpGs using targeted bisulfite sequencing, examine their associations with nearby gene expression in matched samples, and use targeted epigenome editing in relevant cellular models to assess effects on cell-survival and stress-response phenotypes.

Using methylation data, we estimated epigenetic age and related biomarkers with the DNAm Age Calculator [21]. Because the Horvath clock was developed to estimate chronological age from DNA methylation, we first evaluated the correlation between Horvath epigenetic age and chronological age. The two measures were strongly correlated in both MGUS and MM (both *p* < 0.001). The annual change in epigenetic aging was similar between groups (0.85 vs. 0.88 years per chronological year for MGUS and MM, respectively, *p* = 0.89) (Supplementary Figure S4). We did not detect a difference in epigenetic age acceleration (EAA) between the groups (*p* = 0.83; Supplementary Figure S4). With 27 patients with MGUS and 26 patients with MM, the minimum detectable standardized mean difference was approximately 0.79 (Cohen’s *d*) at a two-sided significance level of 0.05 and 80% power. Thus, this result should not be interpreted as evidence of equivalence, and smaller between-group differences cannot be excluded. No statistically significant between-group differences were detected using the alternative epigenetic clock models evaluated (Supplementary Table S8). Because leukocyte telomere length (LTL) has been associated with MM risk and prognosis, and the longer LTL generally observed in healthy AA individuals relative to EA individuals appears attenuated or reversed in MM [22,23,24], we evaluated a DNA methylation-based estimator of telomere length (DNAmTL). DNAmTL decreased with chronological age in both groups. The estimated slope was −0.010 kb/year (SE = 0.007; 95% CI, −0.024 to 0.004) in MGUS and −0.024 kb/year (SE = 0.006; 95% CI, −0.037 to −0.011) in MM. Although the estimated decline was numerically steeper in MM than in MGUS, the age-by-group interaction was not statistically significant (difference in slopes [MM − MGUS] = −0.014 kb/year; 95% CI, −0.034 to 0.005; *p* for interaction = 0.145), providing insufficient evidence that the rate of DNAmTL attrition differed between the groups (Figure 2A). Age-adjusted DNAmTL residuals were lower in MM than in MGUS in an analysis without additional covariate adjustment (*p* = 0.003; Figure 2B). After further adjustment for DNAm-derived blood-cell composition, the adjusted least-squares mean DNAmTL residual was −0.063 in MM and 0.060 in MGUS (adjusted mean difference [MM − MGUS] = −0.123; 95% CI, −0.246 to −0.0001; *p* = 0.050; Figure 2C). Thus, the DNAmTL findings are suggestive but should be considered exploratory. Collectively, these analyses did not detect a statistically significant difference in EAA between MGUS and MM, although smaller differences cannot be excluded given the limited statistical power. The DNAmTL findings were suggestive but should also be considered exploratory.

Our study has several limitations. First, the modest sample sizes limited statistical power, particularly for rare-variant burden testing, and the combination of low variant frequencies and multiple testing increased the potential for both false-positive and false-negative findings. None of the nominal gene-burden associations remained significant after Bonferroni correction; therefore, the *LAMA5* and *PIEZO1* findings should be considered exploratory candidate signals. The absence of an independent validation cohort further limits their reproducibility and generalizability. Second, although patients with MGUS who had no documented progression to MM during at least five years of follow-up provided a clinically meaningful comparator, this classification reflects only the observed follow-up period. MGUS carries an ongoing risk of progression, and some participants may subsequently develop MM. Moreover, the cross-sectional comparison of separate MGUS and established-MM groups cannot determine whether the observed genomic and epigenetic differences preceded MM development, contributed causally to disease development, or arose in association with established disease. Longitudinal studies following patients with MGUS will be required to determine whether any of these features are associated with subsequent development of MM. Third, although all MM samples were collected at diagnosis and before systemic treatment, DNA methylation is dynamic and may be influenced by established disease, systemic inflammation, aging, and circulating blood-cell composition. The MGUS and MM groups were matched on age and sex, and technical batch effects were addressed using ComBat; however, age and sex were not additionally included as covariates in the primary DMP and DMR analyses. Furthermore, methylation was measured in buffy-coat DNA, measured blood-cell composition data were unavailable, and DNAm-estimated blood-cell proportions were not incorporated into the primary DMP or DMR analyses. Some observed methylation differences may therefore reflect variation in circulating leukocyte composition rather than disease-associated epigenetic alterations. Genomic inflation was also not formally evaluated, and residual test-statistic inflation cannot be excluded. The methylation findings should consequently be interpreted as features associated with MM status rather than antecedent susceptibility markers. Fourth, the pathway-enrichment results may be sensitive to CpG-to-gene annotation, gene density, unequal probe representation, and the selected DMP and DMR thresholds, which were not evaluated through a formal sensitivity analysis. The IPA enrichment *p* values were not adjusted for multiple testing, and the analysis of 122 candidate DMRs included regions that did not meet the family-wise error rate threshold for statistical significance. These pathway and upstream-regulator findings should therefore be considered exploratory and hypothesis-generating. Formal multi-omics integration was not performed because of the small sample size; thus, any apparent cross-omics convergence should be considered qualitative. In addition, genetic and epigenetic heterogeneity within populations of AA may not be fully represented in this single-center cohort. Finally, the identified variants, methylation features, and bioinformatically inferred pathways were not functionally validated. These findings require confirmation in larger independent cohorts, longitudinal studies with appropriate adjustment for blood-cell composition, and functional investigations.

In summary, we identified exploratory molecular features associated with MM versus MGUS without documented progression during the observed follow-up period among AA patients. Combined interpretation of the genomic and DNA methylation results suggested potential thematic overlap involving growth and survival signaling, cellular stress responses, DNA damage and repair, apoptosis, and transcriptional regulation; however, this overlap was not evaluated through formal multi-omics analysis. Larger independent cohorts with matched genomic, DNA methylation, gene-expression, and longitudinal clinical data will be required to formally evaluate these relationships.

## Figures and Tables

**Figure 1:**
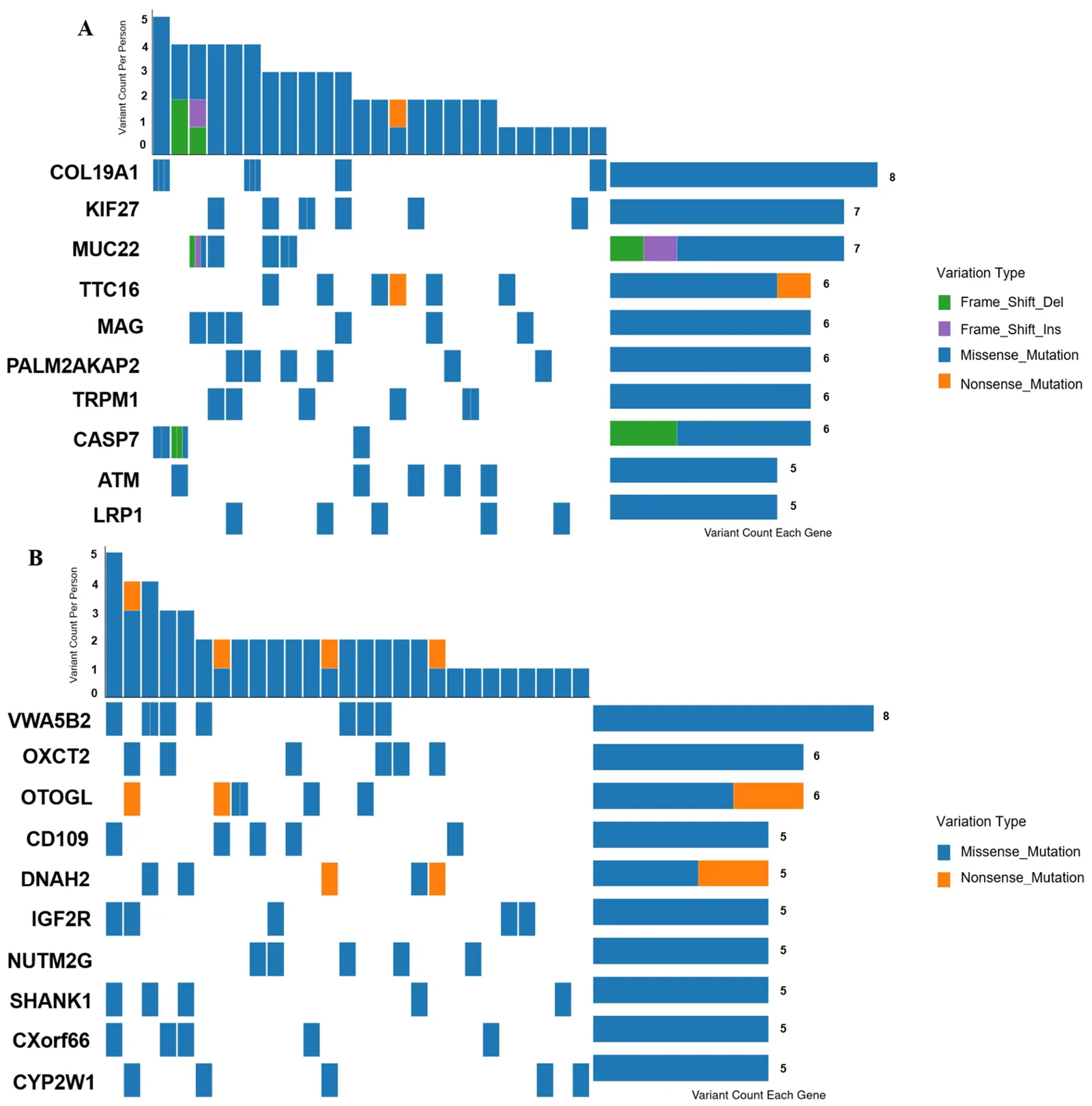
Genes with the highest numbers of qualifying rare coding variant observations detected in only one study group. Genes with at least five qualifying variant observations were ranked by decreasing count, and the 10 genes with the highest counts were displayed for each group. Genes with equal counts were considered tied, and their ordering does not indicate a difference in rank. No additional genes were tied at the 10-gene cutoff. (**A**) Genes containing qualifying variants observed in the MGUS group but not in the MM group. (**B**) Genes containing qualifying variants observed in the MM group but not in the MGUS group. Each column in the central oncoprint represents one deidentified participant; participant identifiers are omitted for visual clarity. Colors indicate variant consequence. The upper bar plot shows the number of qualifying variant observations per participant, and the right bar plot shows the total number of qualifying variant observations per gene. These results are descriptive observations from a small exploratory cohort and do not establish that the displayed variants or genes are specific to MGUS or MM in the broader population.

**Figure 2:**
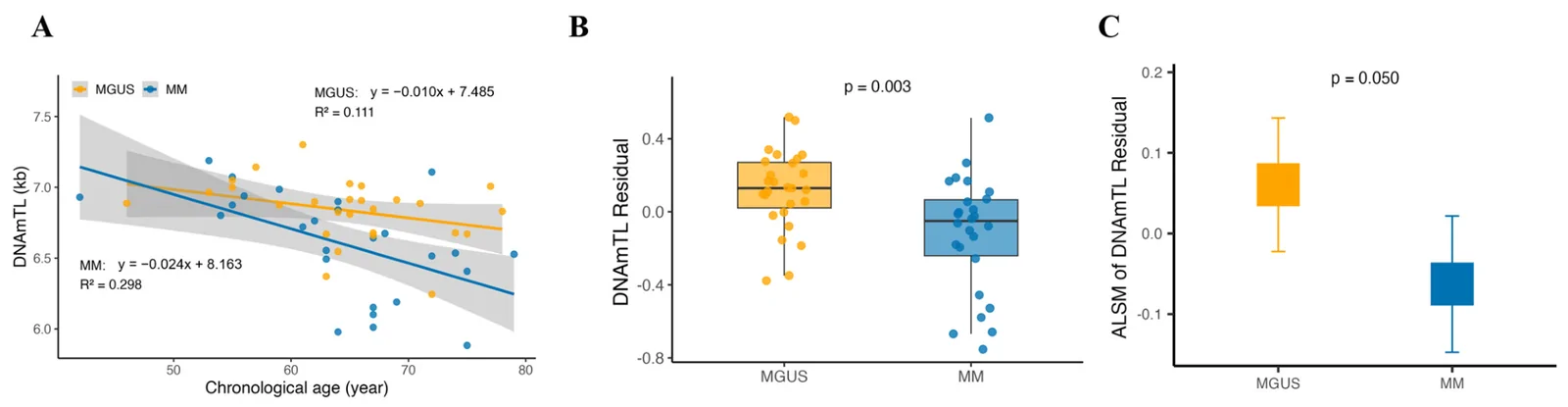
Comparison of DNA methylation-derived telomere length (DNAmTL) between MGUS and MM. (**A**) Linear regression of DNAmTL on chronological age within the MGUS and MM groups. The difference between the estimated slopes was not statistically significant (age × group interaction, *p* = 0.145). (**B**) Comparison of age-adjusted DNAmTL residuals between groups using a two-sample *t*-test without additional covariate adjustment (*p* = 0.003). (**C**) Comparison of adjusted least-squares mean DNAmTL residuals between groups after adjustment for DNAm-derived blood-cell composition (MGUS: 0.06; MM: −0.06; *p* = 0.050). These analyses address different levels of covariate adjustment and should not be interpreted as equivalent tests.

## Data Availability

The individual-level genomic, DNA methylation, and clinical data presented in this study are not publicly available because of participant privacy and ethical restrictions. Deidentified data may be made available by the corresponding author upon reasonable request and subject to institutional approval and applicable data-use agreements.

## Supplementary Materials

The following supporting information can be downloaded at https://www.mdpi.com/article/10.3390/cells15191722/s1, Figure S1: The distribution of DMPs (*q* ≤ 0.05 and |Δβ| ≥ 0.1) and pathway enrichment analysis; Figure S2: The distribution of DMRs (FWER < 1) and pathway enrichment analysis; Figure S3: Top enriched EP300 Pathway; Figure S4: Linear regression lines of Hannum DNA methylation Age (DNAmAge) by chronological age at DNA sampling by MGUS and MM. ALSM: adjusted least squares mean; Table S1: Characteristics of study population; Table S2: Rare variants nominally associated with MM versus MGUS at *p* < 0.05; Table S3: Genes harboring qualifying variants observed in only one study group, restricted to genes with at least 3 observed variants per gene; Table S4: Nominal gene-burden associations with MM versus MGUS at *p* < 0.05; Table S5: Gene set enrichment analysis with FDR < 0.25; Table S6: Differentially methylated positions (DMPs) with FDR *q* ≤ 0.05 and |Δβ| ≥ 0.1; Table S7: Differentially methylated regions (DMRs) with FWER < 1 and *p*.valueArea ≤ 0.025; Table S8: Differences in epigenetic age acceleration (EAA) between MGUS and MM. Refs. [6,25,26,27,28,29,30,31,32,33,34,35] are cited in Supplementary Method.

## Abbreviations

AA, African ancestry; ALSM, adjusted least-squares mean; DMP, differentially methylated position; DMR, differentially methylated region; DNAmTL, DNA methylation-derived telomere length; EA, European ancestry; EAA, epigenetic age acceleration; FDR, false discovery rate; GSEA, gene-set enrichment analysis; LTL, leukocyte telomere length; MGUS, monoclonal gammopathy of undetermined significance; MM, multiple myeloma; PC, principal component; QC, quality control; WES, whole-exome sequencing.
