# Supplementary material for: Germline Genomic and DNA Methylation Differences Between MGUS and Multiple Myeloma Among Patients of African Ancestry
Source: Cells. 2026 Sep 22;15(19):1722. doi: 10.3390/cells15191722 (PMC13663980; doi:10.3390/cells15191722)
Supplement: Supplementary file 1 [file cells-15-01722-s001.zip › Supplementary Methods_R1_final.pdf]

SUPPLEMENTARY METHODS

Study Population

This study was approved by the Institutional Review Board at the Medical College of Wisconsin (MCW) and conducted in accordance with the Declaration of Helsinki. A total of 56 AA patients enrolled from the Froedtert & MCW regional health network were included. Among them, 28 were patients with MGUS who had no documented progression to MM during at least five years of follow-up, and 28 were patients with MM. The median follow-up from MGUS diagnosis was 11.7 years (range, 5–22 years). Diagnoses were confirmed by chart review based on internationally accepted criteria and International Classification of Diseases (ICD) codes. The two groups were matched on age, sex, and immunoglobulin isotype (Supplementary Table 1). Buffy coat samples from all participants were obtained through the MCW Tissue Bank, a College of American Pathologists accredited biorepository. All MM samples were collected at the time of diagnosis and before initiation of systemic treatment.

WES and Variant Calling

Genomic DNA was extracted from buffy coat samples. WES libraries were prepared with 100-200ng of input DNA using the Illumina DNA Prep with Enrichment kit (Illumina, San Diego, CA) following the manufacturer's instructions. Target enrichment (Exome 2.5) was performed, and the resulting libraries were sequenced on the Illumina NovaSeq 6000 platform (2 × 150 bp paired-end) to a mean on-target depth of 100×. Sequence reads were aligned to the GRCh38 human reference genome using the BWA-MEM (v0.7.17), and germline variants were called using the GATK HaplotypeCaller (v4.6.2.0). Standard quality control (QC) filters were applied: including variant quality score ≥20, sequencing depth ≥10, allele balance ≥0.2 for heterozygotes,

and ≤5% missingness per variant and per sample. Low-complexity and segmental duplication regions were excluded. Genetic ancestry was inferred by principal-component analysis of autosomal variants using EIGENSOFT version 7.2.1 using previously generated GWAS data, with populations from the 1000 Genomes Project included as ancestry references.[1] Participants clustering with African ancestry reference populations were included in the analysis, whereas individuals who did not cluster with these populations were excluded. Samples with familial relatedness, extreme heterozygosity, sex discordance or non-African genetic ancestry were excluded. After QC, one MM sample was excluded because of discordance between reported sex and genetically predicted sex, leaving 28 patients with MGUS without documented progression during the observed follow-up period and 27 patients with MM in the WES analysis.

Rare Variant filtering and Functional Annotation

Rare variants (allele frequency ≤1%) were prioritized using African ancestry allele frequencies from the Genome Aggregation Database (gnomAD version 4.1; gnomADe_AFR_AF). Functional annotation was performed using Ensembl Variant Effect Predictor version 115. Qualifying variants included protein-truncating variants (transcript-ablation, splice-acceptor, splice-donor, stop-gained, frameshift, stop-lost, or start-lost variants); missense variants classified as deleterious by SIFT (score ≤0.05) or as possibly or probably damaging by PolyPhen-2 (score >0.446); or variants classified as pathogenic or likely pathogenic by the CharGer (Characterization of Germline Variants) pipeline.[2] An OR criterion was applied to the missense predictions; therefore, variants with discordant SIFT and PolyPhen-2 results were retained if either predictor met its specified threshold. Missense variants that did not meet either

threshold were excluded unless they were classified as pathogenic or likely pathogenic by CharGer.

Single Variant Association, Gene Burden and Gene Set Analysis

For descriptive analyses, a rare coding variant was categorized as “group-observed-only” if it was detected in at least one participant in one study group but not in any participant in the other group. A gene was categorized as group-observed-only if its qualifying rare coding variants were detected in only one study group and no qualifying variant in that gene was detected in the other group. These classifications refer solely to observations within the analyzed cohort and do not imply that the variants or genes are biologically specific to MGUS or MM. Single-variant analyses were restricted to rare, predicted deleterious variants carried by at least three participants. This minimum carrier-count threshold was prespecified before association testing to reduce extremely sparse comparisons, unstable effect estimates, and associations driven by one or two individuals. Each eligible variant was tested for association with the odds of MM versus MGUS using Firth-penalized logistic regression adjusted for age and sex. Gene-burden analyses were performed by collapsing qualifying rare variants within each gene and applying Firth-penalized logistic regression using the R package *brglm2* with Jeffreys-prior bias reduction. Gene-burden results were used to construct a preranked gene list for gene-set enrichment analysis using GSEAPreranked. Analyses evaluated MSigDB version 2026.1 gene-set collections, including Hallmark, positional, Reactome, transcription-factor target, Gene Ontology, Human Phenotype Ontology, oncogenic, and immunologic signatures.[3,4] Because the gene-set enrichment analysis was exploratory, an FDR threshold of $q < 0.25$ was used to identify

candidate pathway-level signals, consistent with conventional GSEA practice. These findings were considered hypothesis-generating rather than definitive evidence of pathway enrichment.

DNA Methylation Profiling

Genome-wide DNA methylation profiling was performed using the Illumina Infinium HumanMethylationEPIC v2.0 BeadChip (Illumina, San Diego, CA), interrogating >850,000 CpG sites. Genomic DNA (250ng) underwent bisulfite conversion using the EZ DNA Methylation Kit (Zymo Research), whereby unmethylated cytosines were deaminated to uracil while methylated cytosines remained unchanged. Bisulfite-converted DNA was amplified, enzymatically fragmented, and hybridized to the array, followed by single-base extension with labeled dideoxynucleotides (ddNTPs). Arrays were stained and scanned according to the manufacturer's protocol.

Raw IDAT files were processed in R using the *minfi* Bioconductor package. Functional normalization was performed using *preprocessFunnorm*. Probe-level SNP annotations were added using *addSnpInfo*, and probes containing annotated SNPs at either the CpG interrogation site or single-base extension site were removed using *dropLociWithSnps*. Sample-level QC included evaluation of methylated and unmethylated signal intensities, β-value density distributions, bisulfite-conversion controls, concordance between reported and methylation-predicted sex, and methylation-profile outliers, with additional quality assessment using Enmix.[5] One DNA sample failed QC during the methylation-array assay. A second sample showed discordance between reported and methylation-predicted sex; this was the same sample excluded from the WES analysis. A third sample was identified as an outlier based on its methylation

profile. These three samples were excluded, leaving 53 samples (27 MGUS and 26 MM) for downstream methylation analyses.

Methylated (M) and unmethylated (U) probe intensities were used to calculate β values as M/(M + U + 100), representing the estimated proportion of methylation at each CpG site. Principal component analysis was used to evaluate major sources of variation and identified two technical batches corresponding to the batch assignments recorded in the sample sheet. ComBat, implemented in the *sva* R package,[6] was applied to the β-value matrix using the recorded technical batch variable. The resulting batch-adjusted β values were used in downstream analyses. Differentially methylated positions (DMPs) between MM and MGUS were identified using *dmpFinder* applied to the batch-adjusted β-value matrix. The MM and MGUS groups were frequency matched on age and sex; therefore, age and sex were not additionally included as covariates in the primary DMP analysis. Effect sizes were calculated as differences in mean β values between groups ($\Delta\beta = \beta_{MM} - \beta_{MGUS}$). CpG sites meeting both $q \leq 0.05$ and $|\Delta\beta| \geq 0.10$ were considered significant. Application of these criteria identified 703 DMPs. Differentially methylated regions (DMRs) were identified using *bumphunter*, with a minimum absolute methylation difference of 0.05 and 1,000 permutations (B = 1,000). Regions with a family-wise error rate (FWER) ≤ 0.05 were considered statistically significant. Only the region associated with *RNF39* met this prespecified significance criterion. For exploratory pathway analysis, we additionally considered 122 candidate regions meeting FWER < 1 and an area-based P value (p.valueArea) ≤ 0.025. These candidate regions were not considered statistically significant DMRs.

Genes annotated to the 703 DMPs and 122 exploratory candidate DMRs were analyzed separately using QIAGEN Ingenuity Pathway Analysis (IPA; application build 9.0; QIAGEN Knowledge Base content version 165342674; analysis performed September 3, 2026). Enrichment of canonical pathways and upstream regulator–target relationships was evaluated using right-tailed Fisher exact tests based on overlap between each input gene set and curated gene sets in the QIAGEN Knowledge Base. Because these analyses were exploratory, no additional multiple-testing correction was applied, and the reported enrichment P values are nominal. The pathway and upstream regulator findings were therefore interpreted as hypothesis-generating.

Genomic inflation was not formally evaluated in the DMP analysis. Given the small sample size and correlated structure of genome-wide methylation measurements, a conventional genomic inflation factor may be unstable; nevertheless, residual test-statistic inflation cannot be excluded. Measured blood-cell composition data were unavailable, and DNAm-estimated blood-cell proportions were not incorporated into the primary DMP or DMR analyses. DNAm-derived cell-composition estimates were used only in the secondary multivariable analysis of DNAmTL. Because methylation was measured in buffy-coat DNA, the observed DMP and DMR associations may partly reflect differences in circulating leukocyte composition between the groups. Therefore, the DMP, DMR, and pathway findings should be considered exploratory and require validation in larger independent studies with appropriate adjustment for blood-cell composition.

Epigenetic Age Measures

Beta values obtained from the DNA methylation array were submitted to the DNAm Age Calculator (https://dnamage.clockfoundation.org) to derive DNA methylation–based measures of epigenetic age and telomere length. Evaluated epigenetic clocks included the original Horvath DNAmAge[7], DNAmPhenoAge[8], DNAmGrimAge[9] and the skin-blood clock[10]. Two additional estimators, GrimAge2 (calibrated to chronological age)[11] and the Zhang epigenetic age[12], were also derived. The relationships between each epigenetic clock and chronological age were examined independently. Pearson's correlation coefficient (r) was used to evaluate the strength of the linear association between epigenetic and chronological age. To compare the annual rate of epigenetic aging between MM and MGUS, separate linear regression models of epigenetic age on chronological age were fitted for each group. Differences in slopes between MM and MGUS were assessed using a multivariable model containing an interaction term between chronological age and disease cohort (chronological age × disease status). Epigenetic age acceleration (EAA) was calculated as the residual from a linear model regressing DNAmAge on chronological age. Crude differences in EAA between MGUS and MM patients were first evaluated using a two-sample *t*-test. To adjust for potential confounding effects, multivariable linear regression models were subsequently fitted with EAA as the dependent variable and disease group (MGUS *vs.* MM) as the primary independent variable, adjusting for estimated blood cell composition derived from methylation data ("CD8.naive", "CD8pCD28nCD45RAn", "PlasmaBlast", "CD4T", "NK", "Mono", and "Gran"). Adjusted least-squares means (ALSMs) of EAA for each cohort were estimated from these adjusted models using marginal means, and pairwise comparisons were performed. Statistical significance across all analyses was determined using two-sided tests ($\alpha = 0.05$), with corresponding confidence intervals derived directly from the fitted models. The same analytical strategy was also applied to DNAmTL.

References

1. Buradagunta CS, Arsang-Jang S, Massat B, Thapa B, Patek V, D'Souza A, et al. Identification of novel loci for multiple myeloma when comparing with its precursor condition monoclonal gammopathy of unknown significance. *Leukemia*. 2024;38(2):383-385.
2. Scott AD, Huang KL, Weerasinghe A, Mashl RJ, Gao Q, Martins Rodrigues F, et al. CharGer: clinical Characterization of Germline variants. *Bioinformatics*. 2019;35(5):865-867.
3. Subramanian A, Tamayo P, Mootha VK, Mukherjee S, Ebert BL, Gillette MA, et al. Gene set enrichment analysis: a knowledge-based approach for interpreting genome-wide expression profiles. *Proc Natl Acad Sci U S A*. 2005;102(43):15545-15550.
4. Liberzon A, Subramanian A, Pinchback R, Thorvaldsdottir H, Tamayo P, Mesirov JP. Molecular signatures database (MSigDB) 3.0. *Bioinformatics*. 2011;27(12):1739-1740.
5. Xu Z, Niu L, Taylor JA. The ENmix DNA methylation analysis pipeline for Illumina BeadChip and comparisons with seven other preprocessing pipelines. *Clin Epigenetics*. 2021;13(1):216.
6. Leek JT, Johnson WE, Parker HS, Jaffe AE, Storey JD. The sva package for removing batch effects and other unwanted variation in high-throughput experiments. *Bioinformatics*. 2012;28(6):882-883.
7. Hannum G, Guinney J, Zhao L, Zhang L, Hughes G, Sadda S, et al. Genome-wide methylation profiles reveal quantitative views of human aging rates. *Mol Cell*. 2013;49(2):359-367.
8. Levine ME, Lu AT, Quach A, Chen BH, Assimes TL, Bandinelli S, et al. An epigenetic biomarker of aging for lifespan and healthspan. *Aging (Albany NY)*. 2018;10(4):573-591.
9. Lu AT, Quach A, Wilson JG, Reiner AP, Aviv A, Raj K, et al. DNA methylation GrimAge strongly predicts lifespan and healthspan. *Aging (Albany NY)*. 2019;11(2):303-327.
10. Horvath S, Oshima J, Martin GM, Lu AT, Quach A, Cohen H, et al. Epigenetic clock for skin and blood cells applied to Hutchinson Gilford Progeria Syndrome and ex vivo studies. *Aging (Albany NY)*. 2018;10(7):1758-1775.
11. Lu AT, Binder AM, Zhang J, Yan Q, Reiner AP, Cox SR, et al. DNA methylation GrimAge version 2. *Aging (Albany NY)*. 2022;14(23):9484-9549.
12. Zhang Q, Vallerga CL, Walker RM, Lin T, Henders AK, Montgomery GW, et al. Improved precision of epigenetic clock estimates across tissues and its implication for biological ageing. *Genome Med*. 2019;11(1):54.
